# Effects of HIV and non-communicable disease comorbidity on healthcare costs and health experiences in people living with HIV in Zimbabwe

**DOI:** 10.4102/sajhivmed.v21i1.1102

**Published:** 2020-09-04

**Authors:** Laston Gonah, Indres Moodley, Khumbulani Hlongwana

**Affiliations:** 1 Health Outcomes Research Unit, Discipline of Public Health Medicine, School of Nursing and Public Health, College of Health Sciences, University of KwaZulu-Natal, Durban, South Africa

**Keywords:** human immunodeficiency virus, non-communicable disease, Zimbabwe, antiretroviral therapy, unemployment, diagnosis and treatment of HIV, ART

## Abstract

**Background:**

The effects of HIV and non-communicable disease (NCD) comorbidities on healthcare costs and health experiences have been documented in most high-income countries. However, little similar data are available for Zimbabwe and most countries in sub-Saharan Africa. Untreated or under-treated NCDs can potentially negate the gains achieved from the control of HIV.

**Objectives:**

The study sought to determine the effects of HIV-NCD comorbidity on healthcare costs, health experiences and treatment options for people living with HIV (PLWH) in Zimbabwe.

**Methods:**

A repeated-measures, quantitative study was conducted at six antiretroviral therapy (ART) sites in the Gweru District of Zimbabwe. Simple random sampling was used to enrol 100 PLWH concurrently diagnosed with hypertension and/or diabetes mellitus (cases). Cases were matched by age, sex and viral load to an equal number of PLWH without hypertension and/or diabetes mellitus (controls). Quantitative data were collected using an interviewer-administered questionnaire at monthly intervals for 6 months. The questionnaire survey sought to compare healthcare costs, health-related experiences and treatment options between cases and controls. Data were analysed using Stata Version 13.1®. A logistic model was used to examine other factors such as demographic, clinical and behavioural data that were assumed to be unchanged over the study period. A random-effects model, including costs and other covariates, was used to compare groups in the final analysis.

**Results:**

Non-communicable disease status was associated with the length of time on ART. Cases spent significantly more on transport (*p* = 0.0001) and medication (adjusted odds ratio [AOR] = 4.4, 95% confidence interval [CI]: 3.2–7.3); spent more days without doing usual daily activities because of sickness (AOR = 4.2, 95% CI: 3.3–7.6) and were more likely to use alternative medication (AOR = 3.4, 95% CI: 2.3–4.6) when compared with controls. Unemployment, female gender, age of 60 years and above, and living in rural areas were associated with failure to purchase prescribed medication.

**Conclusions:**

HIV-NCD comorbidity causes an additional burden to PLWH because of increased transport costs, NCD prescribed medication expenses and more productive days lost due to illness. The success of HIV programmes does not only rely on improving access to the diagnosis and treatment of HIV. Addressing the complications of HIV-related NCDs, and the long-term costs of ART and its occasional potential for harm will be essential if health outcomes in Zimbabweans living with HIV are to be optimised.

## Introduction

Unprecedented donor and government funding to address the HIV and AIDS pandemic resulted in more than 20 million people receiving antiretroviral therapy (ART) by mid-2017, against a total of 36.7 million people living with HIV (PLWH) worldwide.^1^ Whilst ART has markedly increased survival, PLWH have been found to be at greater risk of developing non-communicable diseases (NCDs).^2^

People living with HIV have a threefold increased risk of developing NCDs because of three main reasons: (1) inflammatory and infectious sequelae of HIV infection, (2) the effects of ART treatment itself and (3) finally, the increased risk associated with ageing.^3^ Successful ART roll-out has resulted in most PLWH living longer, possibly driving the onset of NCDs, because of long-term viral infection, accumulation of drug toxins in the body and acceleration of age-related degenerative changes by HIV.^4,5,6^ According to the World Health Organization,^7^ NCDs may be present before HIV infection but could be worsened by HIV or the side effects of some of the antiretroviral drugs.

HIV-NCD comorbidity could have implications for healthcare costs, health experiences and survival compared with HIV alone. Although the effects of HIV-NCD comorbidity on healthcare costs, health experiences and treatment options have previously been documented, especially in high-income countries, this is not true for most sub-Saharan African countries, including Zimbabwe.^2^ Conducting such studies provides valuable information so that targeted intervention strategies can be developed. Studies on HIV-NCD comorbidity are limited to a few cross-sectional surveys that are mainly based on self-reported data without a comparison group.^2,8^

HIV-NCD comorbidity causes an additional healthcare burden. Whilst significant efforts have been made towards HIV control in Zimbabwe, untreated or under-treated NCDs can potentially negate the gains achieved through the national ART roll-out. Currently, no integrated care for HIV-NCD comorbidity is available in Zimbabwe. Despite accessing free services for HIV and AIDS, PLWH have to pay for the treatment of NCDs, mostly through out-of-pocket expenditure, which because of the financial burden, may prevent many patients from seeking care for NCDs.

The study sought to determine the effects of HIV-NCD comorbidity, using hypertension (HTN) and diabetes mellitus (DM) as NCD-tracer conditions, on healthcare costs, health experiences and treatment options in PLWH in the Gweru district of Zimbabwe.

## Materials and methods

### Study design

A repeated-measures, longitudinal quantitative study design was employed.

### Study setting

The study was conducted at six high-volume government ART sites that had the highest number of PLWH and collectively representing over 80% of all PLWH on ART in Gweru district. The six study sites consisted of four urban and two rural sites. The four urban sites are directly administered by Gweru City Council Health Department, whilst the two rural sites are administered by the Ministry of Health and Child Care.

### Study population and participant selection procedures

In this study, HIV patients of either gender, aged ≥18 years, registered for ART programme in the electronic Patient Monitoring System (ePMS) and able to respond to the study questionnaire in Shona, Ndebele or the English language, were considered.

In addition to the age and gender inclusion criteria above, the cases had to be PLWH and concurrently diagnosed with DM and/or HTN. All subjects provided written informed consent to participate in the study. Those with a confirmed diagnosis other than the NCD-tracer conditions of HTN and DM, as required for the study, were excluded. Mentally impaired PLWH and those not on ART were also excluded from the study.

As the ePMS and the Chronic Disease Register are maintained separately, the identification and linkage of PLWH with the tracer NCDs were time-consuming and laborious. A total of 2969 PLWH and comorbid NCDs were identified during a 3-month screening or verification exercise among patients registered for the ART programme. The 3-month period (December 2018 to February 2019) was ideal because all patients were expected to have visited the ART sites for their monthly ART supply. After applying the eligibility criteria, 842 eligible participants from the six sites were identified. Proportional representation, as guided by the total number of eligible participants per site, was followed when selecting participants from rural and urban regions, and from among men and women, within all six sites. Simple random sampling was then performed to obtain a representative sample per site, coming up with a total of the required sample size.

### Sample size and sampling criteria

A sample size of 186 (93 per group) was required to detect a characteristic difference of ± 20% between the two groups with a probability of 95% and power of 80%, assuming 50% in the control group. A 10% assumption was made for missing data and lost to follow-up. As such, a final sample size of 208 (104 per group) was needed to ensure sufficient numbers for analysis.

Two hundred of those providing informed consent, namely 100 pairs, instead of the initially calculated 208 participants or 104 pairs, were enrolled in the study and followed up for 6 months. (It is anticipated that the use of 100 pairs instead of the initially calculated 104 would not significantly affect the study power because 100 per group is still larger than the originally calculated sample size of 93 pairs needed to achieve the given power at the desired standard error.)

The 100 cases were purposively matched by viral load (± 5 copies per milliliter of blood [copies/ml]), age (± 1 years), gender (male, female), distance to ART site (± 2 km) and geographical location (rural, urban) in the ratio of 1 case : 1 control to get a total of 200 study participants.

### Data collection methods

Quantitative data on participants’ demographic profile, disease-related factors, healthcare costs and health experiences were collected using an interviewer-administered questionnaire. Health experiences were measured as the number of days spent by participants without carrying out usual daily activities because of ill health. The questionnaire intended for use was pretested among 10 consenting individuals and adjusted according to pretest findings before final use. Time to complete a questionnaire survey ranged from 15 minutes to 20 minutes per participant. Cases and controls were followed up for 6 months, and data were collected at 1-month intervals. The 6-month follow-up period allowed for repeated measurements, thereby providing sufficient data ‘points’ to determine disease burden, healthcare costs and health experiences. To ensure that participants kept an accurate record of their healthcare costs during the study, participants were requested to keep all receipts of their medical expenses and note down expenditure events within the month. Medications for conditions under study required once-off monthly refills, thereby reducing chances of recall bias.

### Data analysis methods

Data were analysed using Stata Version 13.1®. Descriptive statistics were used to summarise the data for each analysis group. Frequencies and percentages were used for categorical data. Frequencies of numeric data were examined for normality, and means, standard deviation or medians were used as appropriate. A logistic model was used to examine other factors such as demographics, clinical data and behavioural data of the two groups that had been assumed to be unchanged over the study period. A random-effects mixed model including costs and other covariates was used to compare the groups in the final analysis.

### Ethical consideration

Ethics approval for the study was obtained from the Biomedical Research Ethics Committee (University of KwaZulu-Natal) - Ethical Clearance Number: BE086/19, and Ministry of Health and Child Care (from both the District Office in Gweru and the Head Office in Harare). Written informed consent was sought from participants and confidentiality was maintained throughout the study by removal of personal identifiers after entry into the electronic database, and the use of non-identifiable coded numbers. Furthermore, all data were password protected in the electronic subject-storage database.

## Results

### Demographics of study participants

Equal numbers of cases and controls (100 participants each) were enrolled into each arm. The age of the participants ranged from 33 to 80 years, with a mean age of 57 (SD ± 10.79) years. The proportion of female participants was greater than that of male participants (Table 1). The majority were unemployed at the time of the study, with no significant difference in financial status between cases and controls. Mean monthly income per participant was $17.56 (SD ± 28.37), and grants received from relatives had a mean of $6.66 (SD ± 24.26). Thirty-eight per cent of participants had viral loads of 0 copies/mL (undetectable viral load), whilst 34% had viral loads of below 20 copies/mL (Table 1). Average duration on ART at baseline for cases was 10.22 years (SD ± 3.165) and was 4.32 years (SD ± 2.12) among controls.

**TABLE 1:** Baseline demographic study data.

Variable	Cases (*N* = 100)		Controls (*N* = 100)	
	Frequency (*n*)	%	Frequency (*n*)	%
**Sex**				
Male	41	41.0	41	41.0
Female	59	59.0	59	59.0
**Age (years)**				
30–39	7	7.0	6	6.0
40–49	21	21.0	22	22.0
50–59	28	28.0	27	27.0
60–69	34	34.0	35	35.0
70 and above	10	10.0	10	10.0
**Marital status**				
Married	45	45.0	46	46.0
Widowed	41	41.0	30	30.0
Divorced	14	14.0	23	23.0
Unmarried or single	0	0.0	1	1.0
**Level of education**				
Primary	59	59.0	59	59.0
High school	33	33.0	33	33.0
Tertiary	8	8.0	8	8.0
**Employment status**				
Formally employed	9	9.0	9	9.0
Informally employed	29	29.0	29	29.0
Unemployed	62	62.0	62	62.0
**Area of residence**				
Urban	55	55.0	55	55.0
Rural	45	45.0	45	45.0
**Mortality (deaths)**	**3**	**3.0**	**0**	**0.0**
**Viral load (copies/mL)**				
0 (undetectable viral load)	38	38.0	38	38.0
< 20	34	34.0	34	34.0
20–50	19	19.0	19	19.0
51–100	9	9.0	9	9.0
**Health insurance status**				
Yes	26	26.0	25	25.0
No	74	74.0	75	75.0
**Alcohol intake**				
Yes	4	4.0	9	9.0
No	96	96.0	91	91.0
**Smokers**				
Yes	1	1.0	5	5.0
No	99	99.0	95	95.0

Over 98% of study participants were followed for the entire 6 months of the study with *n* = 3 deaths among the cases, and no loss to follow-up. In the analysis, the mixed-effects model was employed to deal with deaths, and all observations that contributed to the results were measured up to the point of the participant’s death. Table 1 shows demographic and other key characteristics of study participants.

### The non-communicable disease study group, data and outcomes

Table 2 shows that the proportion of cases with HTN alone was greater than that with DM alone, both at baseline and at the end of the study. None of the controls had a diagnosis of any NCD at baseline. The presence of ‘new’ NCDs in cases and controls at the 6-month follow-up census is shown in Table 2. Three (*n* = 3) deaths (HIV or comorbid disease related) were recorded among cases during the study period.

**TABLE 2:** Disease-related information among cases and controls.

Condition	Cases at baseline (*n* = 100)		Cases at end of study (*n* = 97)		Controls at end of study (*n* = 100)	
	Frequency	%	Frequency	%	Frequency	%
Hypertension	84	84.0	88	90.7	4	4.0
Diabetes mellitus	38	38.0	39	40.2	4	4.0
Diabetes and hypertension	22	22.0	29	29.9	0	0

Note: Numbers in the columns do not total 100% in view of the presence of overlapping disease in the participants.

During the follow-up period, three new diagnoses of DM were recorded in the case group, whereas four new diagnoses of DM were recorded in the control group. Again, four new cases of HTN were diagnosed in the case group, and four new cases in the control arm. Other new diagnostic conditions recorded during the study were peptic (gastrointestinal tract) ulcers (four among cases and two among controls), asthma (two among cases and none among controls), arthritis (three among cases and one among controls) and cataract (one among cases and none among controls).

**FIGURE 1:**
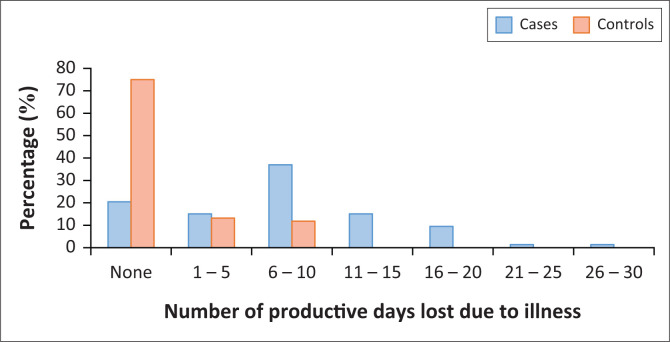
Average number of days spent without doing usual daily tasks because of sickness.

The average monthly number of productive days lost because of sickness was compared between cases and controls. On average, cases spent 11 more days unable to do usual daily activities because of self-reported illness when compared with controls (Figure 1).

### Out-of-pocket expenses

**FIGURE 2:**
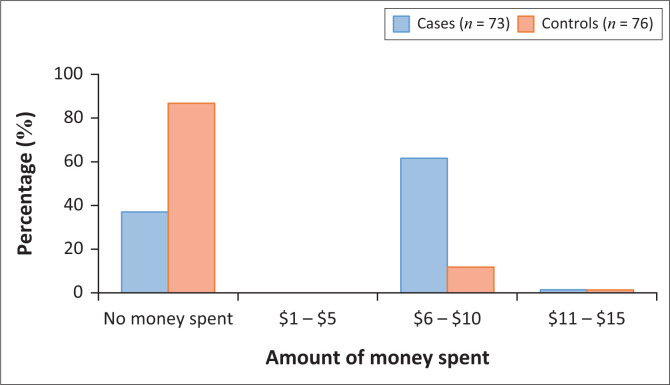
Average monthly transport expenses.

Average monthly amount of money spent on transport by participants for travelling to access healthcare or medication was compared between cases and controls. In general, 52% more cases spent money on transport compared with controls (Figure 2).

The participants’ ability to purchase DM and antihypertensive medications was assessed. More than 68% reported an inability to purchase prescribed medication for their conditions during the study period (Table 3).

**TABLE 3:** Summary of participants’ ability to purchase medications.

Medication purchased	Cases		Controls	
	Frequency (*n*)	%	Frequency (*n*)	%
**Hypertension**				
Yes	28	31.8	2	50.0
No	60	68.2	2	50.0

**Total**	**88**	**100.0**	**4**	**100.0**
**Diabetes mellitus**				
Yes	7	17.9	1	25.0
No	32	82.1	3	75.0
**Total**	**39**	**100.0**	**4**	**100.0**

The average monthly amount of money spent on prescription medication for HTN and DM was assessed for the study period in both the cases and the controls (Table 4). A significant number of cases did not have money to purchase their prescribed medication. Only 14.8% reported consistently getting antihypertensive medication for free at their local health centre during the study period. None of the cases and controls reported receiving free diabetic medication from a health facility during the study period.

**TABLE 4:** Average monthly amount of money spent on medication ($).

Variable	Cases		Controls	
	Frequency (*n*)	%	Frequency (*n*)	%
**Diabetes mellitus**				
None	31	79.5	3	75.0
≤ $10	3	7.7	0	0.0
$11–$15	4	10.3	0	0.0
> $15	1	2.5	1	25.0

**Total**	**39**	**100**	**4**	**100**
**Hypertension**				
None	53	60.2	1	25.0
≤ $10	29	33.0	1	25.0
$11–$15	3	3.4	2	50.0
> $15	3	3.4	0	0.0
**Total**	**88**	**100**	**4**	**100**

Concerning the use of traditional medicines, more cases (83.4%) reported using traditional medicines for the treatment and management of ill health compared to the control group (5.3%). Similarly, the majority (> 73.0%) reported using traditional medication for the management of DM and/or HTN in the case group, than in the control group who developed HTN or DM (see Table 5).

**TABLE 5:** Use of traditional medicines for treatment.

Variable	Cases		Controls	
	Frequency (*n*)	%	Frequency (*n*)	%
**Hypertension**				
Yes	65	73.9	3	75.0
No	23	26.1	1	25.0

**Total**	**88**	**100.0**	**4**	**100.0**
**Diabetes mellitus**				
Yes	30	76.9	4	100.0
No	9	23.1	0	0.0
**Total**	**39**	**100.0**	**4**	**100.0**

### Usual diet using the 24-hour food recall method

**FIGURE 3:**
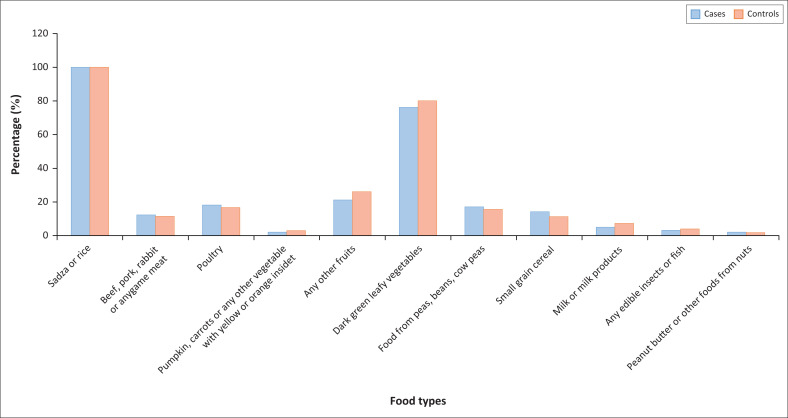
Common foods consumed by participants: 24-h food recall.

Participants were asked to name the type of food they had consumed in the previous 24 hours prior to the questionnaire survey, and subsequently for all the six (6-Xs) monthly visits. The usual diet for more than 80% of both cases and controls was carbohydrate staples, such as rice and maize meal (*sadza*), which often included green leafy vegetables. Less commonly consumed foods, consumed by less than 30% of the participants, were meat (all types), fruits, cereals, milk and milk products and legumes (Figure 3).

### Analytical statistics of demographic and personal characteristics according to non-communicable diseases status and type, days spent without usual daily tasks and the ability to purchase non-communicable disease medication

Our analysis compared healthcare costs and the health-related experiences of cases versus controls. The parameters that were assessed included the NCD diagnosis, the number of days spent without doing usual daily tasks or activities and the ability to purchase prescribed medication. Covariates included demographic variables such as age, sex, employment status, marital status, health insurance status, use of alternative medication, transport cost, viral load, duration on ART, distance from the ART centre, smoking and alcohol consumption.

### Non-communicable disease status in relation to the use of alternative medication, transport costs and hospitalisation

Compared with the control group, the presence of target NCDs in the case group was associated with a longer duration on ART, that is, > 5 years, after controlling for other factors (*p* = 0.0023). As cases were matched to controls by age, sex, employment status, viral load and area of residence, these covariates were not associated with NCD status between the two groups. However, subgroup analysis of the case group indicated that two or more NCDs were more common in female cases compared with male cases (*p* = 0.0021).

Use of alternative or traditional medicines was compared between cases and controls. Cases were significantly more associated with use of alternative or traditional medication compared with controls (adjusted odds ratio [AOR] = 3.4, 95% confidence interval [CI]: 2.3–4.6, *p* = 0.0001).

Having an NCD was significantly associated with higher monthly transport costs (*p* = 0.0001). Rural cases in particular were associated with higher transport costs compared with urban cases, urban controls and rural controls (*p* = 0.0041). The average distance to the ART centre was 15 km for rural cases as compared with 7 km for urban cases. The usual source of NCD medication (HTN and DM) for the majority of cases (over 85%) was private pharmacies, except for only 14.8% of hypertensive patients whose usual source was other public health facilities.

Cases, whether diagnosed with DM, HTN or both, were more likely to be hospitalised compared with controls (AOR = 2.4, 95% CI: 1.2–3.3). The average number of hospitalisation days in cases was 5 days compared with 1 day in controls.

### Days spent without usual daily tasks or activities because of illness

To assess the effect of NCDs on productivity, average monthly number of days spent without doing usual daily activities because of illness was compared between cases and controls. Cases were more likely to spend more days without doing usual daily activities compared with controls (AOR = 4.2, 95% CI: 3.3–7.6, *p* = 0.0000).

Days spent without doing usual daily tasks or activities were associated with female gender, inability to purchase medication and unemployment status. Women spent more days without usual daily activities when compared with men in both cases and controls (*p* = 0.0031). Again, the inability to purchase prescribed medication was associated with more days spent without doing usual daily activities because of illness in both cases and controls (*p* = 0.0023). Unemployed participants spent more days without doing ‘usual’ daily activities as compared with formally employed and informally employed participants (*p* = 0.001). Subgroup analysis of cases showed that cases with more than two NCDs spent more days without doing usual daily activities (AOR = 2.1, 95% CI: 1.3–4.4).

### Ability to purchase non-communicable disease medication

Not all patients could afford their prescribed medication. Therefore, simply estimating costs on the basis of monthly prescriptions would not have represented the actual situation on the ground. Participants – cases and controls – were asked to indicate monthly medication expenses and to record for the duration of the study, which medicines had not been purchased each month. This inability to purchase prescribed medication was used in this study as an adjunctive measure of the effect of NCDs on healthcare costs in PLWH.

Cases were less likely than controls to be able to purchase medication for ill health. Inability to purchase medication was associated with having an NCD (AOR = 4.4, 95% CI: 3.2–7.3). Among the cases, the inability to purchase NCD medication was associated with the number of NCDs per patient, sex, age and employment status. Cases with two or more NCDs were more unlikely to purchase medication compared with those with one NCD (*p* = 0.000). Female participants were less likely than male cases to afford monthly medication requirements (*p* = 0.0011). Participants living in rural areas and those in the age categories 60–69 years and ≥70 years were similarly less likely to afford medications, than urban participants and those aged <60 years (*p* = 0.0031, 0.0001 and 0.0000, respectively). Formal employment was significantly associated with the ability to purchase medication, after adjusting for potential confounders (*p* = 0.000). Estimated average monthly expense on medication among cases was $12 compared with $1 among controls, considering only those who were able to purchase all the prescribed medication.

### Assessment of usual diet

There was no significant difference in usual diet between cases and controls, using the 24-hour food recall method. The usual diet for both cases and controls was carbohydrate staples and green leafy vegetables, with less protein sources of food. Employment status was significantly associated with consuming a balanced diet, rich in all the required nutrients, in both cases and controls (*p* = 0.0003). Consumption of a balanced diet was not associated with other demographic characteristics and NCD status.

## Discussion

The study compared healthcare costs, health experiences and care-related outcomes in PLWH diagnosed with HTN and/or DM with a matched control group of PLWH without NCDs. This study observed numerous important findings of relevance to PLWH in Africa (Zimbabwe) who are on ART and virally suppressed:

- The concurrent presence of comorbid disease is a function of time on ART.
- HIV-NCD comorbidity undermines the goals of HIV treatment, which is to control the virus (HIV) and promote wellness: (1) expenses increase, for example, medication and travel costs; (2) impaired management of comorbid conditions, where over 68% of the case group were unable to afford DM or HTN medication, which were not supplied free-of-charge; (3) increasing vulnerability to non-evidence-based health options and (4) HIV-NCD comorbidity results in greater risk of morbidity and mortality.
- Women bear the greater burden of comorbid disease, and experience greater ‘disability’ – unable to do usual daily tasks.
- Rural citizens and the elderly appear to experience a greater negative impact of economic hardship from comorbid conditions.

In general, controls, namely, virally suppressed PLWH without HTN, DM or both, were associated with lower average monthly expenses on prescription medication and spent fewer number of days without carrying out usual daily activities or tasks because of illness, compared with virally suppressed PLWH with HTN and/or DM. In Zimbabwe, most PLWH access ART free-of-charge in public health facilities.^9^ However, medication for the NCDs is usually not available free-of-charge. These patients have to access medication largely through out-of-pocket expenses. Ability to pay for NCD medication, therefore, becomes a major determinant for access to medication, which results in poor management of the NCDs and the generally observed higher number of productive days lost because of illness. This has potential to impact negatively on the gains achieved so far in controlling HIV through ART.

Unemployment, gender, age and distance to a healthcare facility are well-known key determinants of health.^10^ In this study, significant associations were found between these key determinants of health and the ability to purchase prescribed medication, as well as days spent without carrying out usual daily activities because of illness. This finding can be explained in part by the fact that the majority of employed participants in the study were men, below the age of 60 years. Given that antihypertensives and diabetic medications were largely accessible through out-of-pocket payment, employment enables those with NCDs to purchase the prescribed medications and take control of their health. In patriarchal societies, such as Zimbabwe, opportunities for income and control of resources are biased towards the male gender compared with females.^11^ The female gender and unemployment are inextricably linked together in such societies, predisposing unemployed female participants to negative health outcome, as observed in this study.

On the other hand, the lower proportion of male participants and the fewer number of days these male participants spent without undertaking usual daily activities because of illness, compared with female participants, needs further inquiry. Observed findings could have been influenced in part by poor health seeking behaviours, which are commonly associated with the male gender.^12^ Because of their masculinity, characteristic of men, participants might deliberately under-report the number of days they spent without carrying out usual daily activities. Perhaps, the perception of serious ill health in men might be different from that in women.^12,13,14^

In general, cases were found to spend more days without undertaking usual daily activities because of illness compared with controls. A study conducted in Namibia found similar results, where sickness because of HTN and DM was the top cause of absenteeism among workers in the formal sector.^15^ Cases aged 60–69 years and ≥ 70 years were more likely to spend more days without carrying out usual daily activities or tasks because of illness. They were more at risk of not being able to purchase NCD medication, compared with other age categories, and compared with similar age categories in controls, after controlling for potential confounders.

Productivity days lost because of illness are linked with lower income or loss of employment or limited opportunities for work, which translates to lack of income to purchase the needed medication. A study conducted in USA^16^ found out-of-pocket medication costs to be a significant challenge faced by older adults with DM, often forcing them to cut back on medication use, forgoing food or other basic necessities or borrowing money. The age group ≥60 years is usually associated with retirement and diminished ability and opportunities for employment.^17^ When medication for NCDs has to be purchased through out-of-pocket payments, this age group is at risk because of inability to afford medication and consequently face a greater risk for the observed poorer health outcomes.

Having two or more NCDs was markedly associated with more days spent without undertaking usual daily activities and more money spent on medication, compared with cases with one NCD. Having more than one NCD naturally translates to more money needed to purchase medication, thus increasing the possibility of inability to purchase the required medication, especially given the fact that the majority of the participants were either unemployed or had an unstable source of income. Without access to prescribed medication to manage their health, patients would be predisposed to poorer health outcomes as indicated by productive days lost because of illness observed in this study. Patients were more likely to opt for alternative medication from informal sources to manage their health in the face of limited or no income to purchase prescribed medication.

Use of alternative medication was commonly reported among cases as compared with controls. However, use of alternative medication was not associated with positive improvements on days spent without undertaking daily activities because of sickness. Use of alternative medication may have been influenced to some extent by the cases’ inability to purchase NCD medication, forcing patients to resort to cheaper alternatives to manage ill health. This could be assumed, given that the controls did not report use of alternative medication for management of HIV other than ART. Common alternative medication included home remedies and traditional herbs that are readily available at lower costs. Toxicity, maximum safe dosage and clinical efficacy for these treatment options are usually not known, thus potentially predisposing the users to other unknown negative health consequences.^18^ Future studies should assess the various types and sources of alternative medications commonly used by this patient population to gain a better understanding about this issue.

Higher out-of-pocket medication and transport expenses faced by cases can push patients to employ various coping mechanisms, including cutting back on medication use to adopt traditional medication or forgoing other basic necessities including food.^16^ Finally, we need further enquiry to better understand challenges experienced by people with HIV-NCD comorbidity (especially the difficulties faced in accessing medication and care) and the coping mechanisms that they employ in dealing with these challenges.

### Study limitations

- Measurement of the outcome variable, ‘number of days spent by participants without doing usual daily activities or tasks’, was based on self-reported responses by participants and not objectively ascertained. Although effort was put in corroborating individual responses with further follow-up questions, there is a possibility that variations in personal characteristics could have affected how participants responded to ill health (to do or not to do usual daily tasks). Again, recall bias cannot be ruled out in the measurement of this variable. However, we believe that self-reported episodes of serious sickness within a month are less likely to vary significantly from the actual episodes.
- Adherence to ART use in this study was not assessed, but rather assumed, based on satisfactory HIV viral load measurements of participants. There is a possibility that the observed health experiences measured in this study might not have been sorely because of inability to purchase NCD medication alone, but because of interaction with non-compliance to NCD prescribed medication or use of traditional medication for NCDs that was common among cases in this study.
- Survival was not assessed in this study to compare survival rates between cases and controls. Whilst the 6-month follow-up was adequate to assess trends in health expenses and health experiences, it was not long enough for survival analysis to be performed. Survival analysis would have required longer a follow-up period of 5–15 years, which would not have been feasible for this study.
- Representativeness of our research data to other African countries with stable economies might be limited, in part, because of the economic crisis faced by Zimbabwe, which makes it unique in sub-Saharan Africa.

## Conclusion

HIV-NCD comorbidity causes an additional burden to PLWH because of increased transport costs, NCD prescribed medication expenses and more productive days lost because of illness. Access to antihypertensives and diabetic medications is influenced by interplay of the key determinants of health, which include income, gender, age and geographical location. The success of HIV programmes does not only rely on improving access to the diagnosis and treatment of HIV. Addressing the complications of HIV-related NCDs and the long-term costs of ART and its occasional potential for harm will be essential if health outcomes in Zimbabweans living with HIV are to be optimised.
